# How to Personalize General Anesthesia—A Prospective Theoretical Approach to Conformational Changes of Halogenated Anesthetics in Fire Smoke Poisoning

**DOI:** 10.3390/ijms25094701

**Published:** 2024-04-25

**Authors:** Flavius Nicușor Truicu, Roni Octavian Damian, Mihai Alexandru Butoi, Vlad Ionuț Belghiru, Luciana Teodora Rotaru, Monica Puticiu, Renata Maria Văruț

**Affiliations:** 1 Emergency Medicine and First Aid Department, Faculty of Medicine, University of Medicine and Pharmacy of Craiova, 200349 Craiova, Romania; flavius.truicu@rmu.smurd.ro (F.N.T.); ronidamian@yahoo.com (R.O.D.); mihai.butoi@rmu.smurd.ro (M.A.B.); vlad.belghiru@rmu.smurd.ro (V.I.B.); 2 Emergency Medicine and First Aid Department, Faculty of Medicine, University of Medicine and Pharmacy “Vasile Goldiș” Arad, 310025 Arad, Romania; 3 Research Methodology Department, Faculty of Pharmacy, University of Medicine and Pharmacy of Craiova, 200349 Craiova, Romania; renata.varut@umfcv.ro

**Keywords:** mass fire events, general anesthesia, smoke inhalation, hydrochloric acid, halogenated anesthetics, computational chemistry, personalized therapy, hemoglobin blockade, myoglobin blockade

## Abstract

Smoke intoxication is a central event in mass burn incidents, and toxic smoke acts at different levels of the body, blocking breathing and oxygenation. The majority of these patients require early induction of anesthesia to preserve vital functions. We studied the influence of hemoglobin (HMG) and myoglobin (MGB) blockade by hydrochloric acid (HCl) in an interaction model with gaseous anesthetics using molecular docking techniques. In the next part of the study, molecular dynamics (MD) simulations were performed on the top-scoring ligand–receptor complexes to investigate the stability of the ligand–receptor complexes and the interactions between ligands and receptors in more detail. Through docking analysis, we observed that hemoglobin creates more stable complexes with anesthetic gases than myoglobin. Intoxication with gaseous hydrochloric acid produces conformational and binding energy changes of anesthetic gases to the substrate (both the pathway and the binding site), the most significant being recorded in the case of desflurane and sevoflurane, while for halothane and isoflurane, they remain unchanged. According to our theoretical model, the selection of anesthetic agents for patients affected by fire smoke containing hydrochloric acid is critical to ensure optimal anesthetic effects. In this regard, our model suggests that halothane and isoflurane are the most suitable choices for predicting the anesthetic effects in such patients when compared to sevoflurane and desflurane.

## 1. Introduction

Mass fire incidents have become increasingly prevalent in recent times, posing a significant threat to public safety. The complex nature of these events leads to a high number of casualties and often to severe and multifaceted injuries. The primary risks associated with such incidents include polytraumas, burns, and, last but not least, smoke intoxication, which can all result in life-threatening consequences.

It has been proven that a large percentage of deaths caused by mass burning events are due to multifactorial poisoning with combustion products [CO (carbon monoxide), CO_2_ (carbon dioxide), HCN (hydrogen cyanide), HCl, HBr (hydrogen bromide), Nox (nitrogen oxides) and SOx (sulfur oxides)] [1,2,3]. The composition of toxic smoke differs quite a lot depending on the type of combustible materials or the environment in which the fire occurs. Plastic materials, widespread today, are among the main generators of toxic combustion products, releasing high concentrations of intoxicants such as hydrocyanic acid or hydrochloric acid [4]. The concentration of hydrochloric acid released during a fire depends on the weight of the burned plastic. Polyvinyl Chloride (PVC) has been shown to turn approximately 50% of its weight into hydrochloric acid through pyrolysis [5]. The effects of hydrochloric acid poisoning on organisms depend on its concentration; values as low as 50 parts per million (ppm) can cause upper and lower respiratory tract irritation, with lethal effects observed in animal experiments at values exceeding 800 ppm [6]. One of the determining factors of poisoning with fire smoke products is the blocking of hemoglobin and myoglobin. Although the dynamics of hemoglobin blockade by carbon monoxide and cyanide are well understood [7,8,9,10], the behavior of hydrochloric acid towards it remains unclear. At the same time, myoglobin blockade, which is accompanied by the blocking of hemoglobin in these conditions [11], can also be an impact factor in perturbing tissue oxygenation, muscle metabolism in general, and myocardial metabolism in particular, being crucial for rhabdomyolysis infiltration, multiple organ dysfunction syndrome, and patient prognosis [12]. The patients resulting from these extensive events, regardless of the prevalence of their injuries (blast, severe smoke poisoning, burns, polytrauma), frequently require advance support or vital function stabilization, even before evacuation from the scene, during the transfer, or in the emergency departments, pain management, sedation, or general anesthesia [13]. General anesthesia is part of the advanced management for burn injury surgical procedures, smoke poisoning, and damage control for major associated trauma, being frequently extended over weeks or months, requiring adaptation of induction and maintenance protocols to the constantly changing status of patients [14,15,16].

Halogenated volatile anesthetics serve as a valuable tool in emergency departments due to their unique properties, such as anti-inflammatory and bronchodilator effects, favorable pharmacokinetic and pharmacodynamic characteristics, and ease of administration [17]. Halothane, isoflurane, sevoflurane, and desflurane are volatile anesthetics that are widely used in general anesthesia maintenance, particularly for hypnosis. These anesthetics are also used during anesthesia induction, even in pediatric patients, and have similar effects to propofol induction. It is known that at these ages, especially under three years, there are emotional and practical aspects related to vascular access (difficult intravenous access), which can be overcome by using the inhalation route [18]. There are varying conclusions among several studies regarding the benefits, risks, and adverse effects of using propofol in pediatric patients, but the conclusions drawn from these studies are often contradictory [19]. Some studies suggest that propofol use may lead to neurotoxicity and neuroapoptosis [20,21,22], whereas others indicate that it may depress ventilation and the body’s response to hypoxia [23,24,25]. Therefore, further research is necessary to determine whether using halogenated anesthetics in these situations may improve outcomes.

From a structural point of view, they are halogenated ethers containing chlorine (Cl), fluorine (F), and bromine (Br) (halothane—C_2_HBrClF_3_), chlorine and fluorine (isoflurane—C_3_H_2_ClF_5_O), and only fluorine (desflurane—C_3_H_2_F_6_O and sevoflurane—C_4_H_3_F_7_O). The substitution of compounds with fluorine induces changes in their physicochemical properties [26], which confers increased resistance to metabolism and enhances their stability. The efficacy of an inhaled anesthetic is quantified by the minimum alveolar concentration (MAC), which denotes the minimum concentration of a halogenated agent in the alveolus at 1 atm, where 50% of the subjects do not exhibit movement in response to a painful stimulus. The MAC value is represented as a percentage or atm unit and is defined as the halogenating agent alone in pure oxygen. The potency of the drug is inversely proportional to the MAC value, where a lower value indicates greater effectiveness of the anesthetic agent [27].

Halogenated anesthetics are closely associated with chlorofluorocarbons (CFCs). CFCs release free chlorine, volatile anesthetics being also capable of releasing free chlorine [28].

This research puts forward a hypothesis that inhalation of fire smoke that contains a significant amount of hydrochloric acid limits the capacity of hemoglobin and myoglobin to bind and carry oxygen [29], ultimately impacting cellular metabolism. Additionally, different halogenated agents belonging to different generations can interact to modify myoglobin and hemoglobin, resulting in different conformations that can affect stability and reactivity compared to the known ones. This can result in different therapeutic behaviors for anesthetic agents.

In theoretical chemistry, computational chemistry plays an essential role in predicting interactions and reactions that may not be observable in practice [30]. Doing so can forecast clinical effects without patient exposure, making it a valuable and forward-looking tool for researchers and practitioners. Recent advancements in computational techniques have catalyzed significant progress in the field of drug discovery, particularly through molecular dynamics simulations. Two distinct studies contribute to this evolving landscape by demonstrating innovative approaches in system-level simulations and structural analysis of biomolecules. The first study, employing the heterogeneous CPU (central processing unit) + GPU (graphics processing unit)-enabled simulations within the DFTB (density-functional based tight binding) framework, addresses the computational challenges associated with large-scale molecular dynamics of biological systems. By integrating CPU and GPU resources, this approach not only enhances the simulation speed but also maintains a low power consumption, crucial for exascale computing initiatives. This methodology is particularly effective in handling the diagonalization of the Hamiltonian matrix, a frequent process in molecular dynamics trajectories of large systems such as the explicitly solvated HIV (Human Immunodeficiency Virus) protease, which encompasses 3974 atoms. This example highlights the potential of heterogeneous computing in achieving detailed, quantum-based molecular dynamics simulations at an unprecedented scale [31].

The second study focuses on the structural dynamics of the HIV-1 protease, a key enzyme in the HIV replication process, utilizing a combination of classical molecular dynamics and quantum mechanical methods. Through an innovative integration of these techniques, the study reveals intricate details about the binding interactions and dynamics within the protease. By combining empirical and quantum mechanical insights, the approach provides a comprehensive understanding of the enzyme’s mechanism, which is crucial for the rational design of inhibitors. The use of advanced computational methods allows for the exploration of the dynamic conformational changes of the protease and its interaction with potential inhibitors, providing valuable insights that are not accessible through traditional static computational methods [32].

These studies underscore the transformative impact of advanced computational strategies in drug discovery. They illustrate how leveraging heterogeneous computing and integrating multiple simulation methodologies can provide deeper insights into complex biological phenomena, thereby accelerating the development of effective therapeutic agents.

## 2. Results

**Figure 1:**
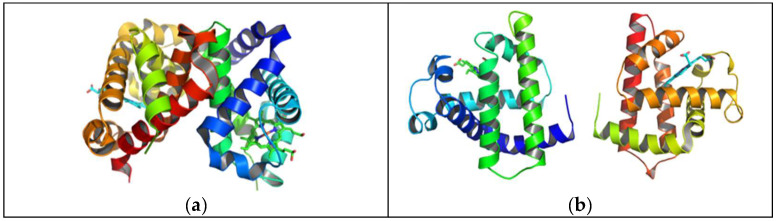
Optimized molecular structure of hemoglobin (**a**) and myoglobin (**b**).

In this study, the molecular geometries of the targets were, by default, modeled and optimized using the semi-empirical PM6 Gaussian program suite (Figure 1), which made it possible to obtain data on the electronic structure of the studied substances (molecular electronic levels, electronic population, dipole moment, net electric charges of atoms, energy partitions by type of chemical bonds or interactions, bound order, free valence, etc.).

### 2.1. Conductor-like Polarizable Continuum Model (C-PCM) and Molecular Electrostatic Potential (MEP) Maps of Halogenated Anesthetics

**Figure 2:**
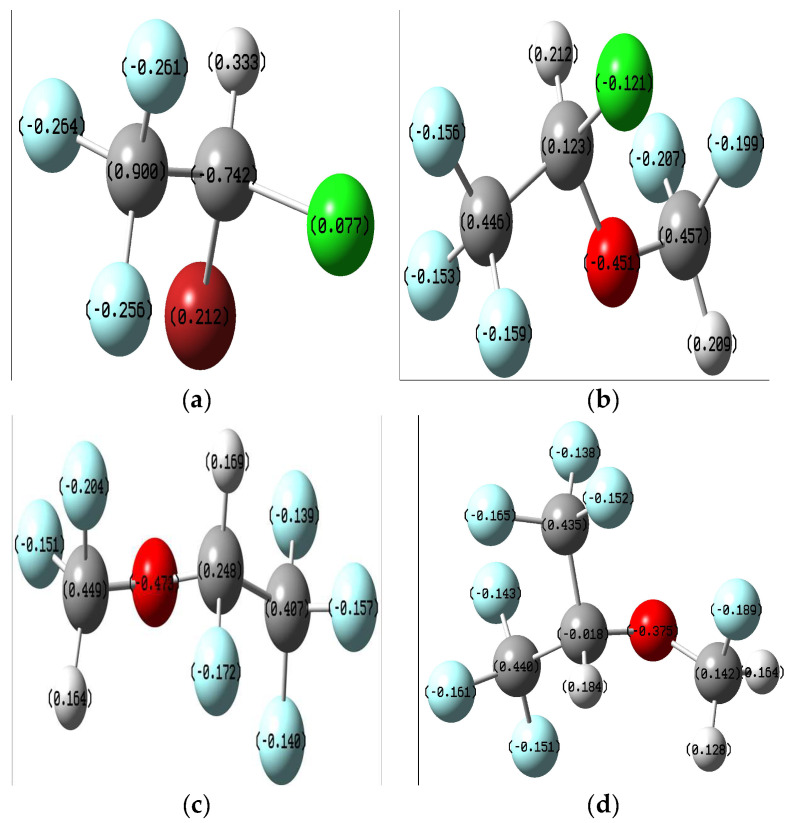
Mulliken charge distribution on the optimized ligands: (**a**) Halothane; (**b**) Isoflurane; (**c**) Desflurane; (**d**) Sevoflurane.

The data presented in the figure below (Figure 2) regarding the partial charges of the atoms in the molecules resulting from the Mulliken popular scheme show the existence of several atoms with a great tendency to donate electrons to the biological receptor: F, Cl, Br, and O. Therefore, it can be assumed that chemical interactions of the ligands with the receptor are possible through these centers.

Due to the number of polar bonds and the possibility of O-H-halogen bonds formation, the anesthetic structures may be sensitive to the solvent interaction. Structures were also optimized in water using the C-PCM implicit solvation model.

**Figure 3:**
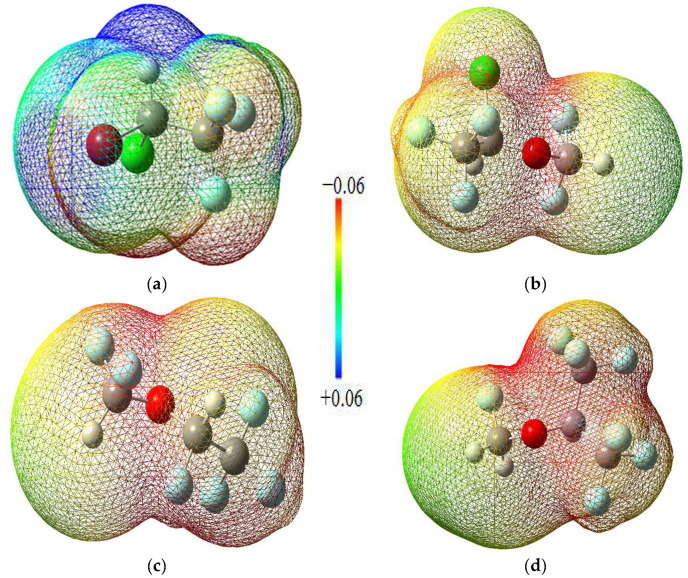
MEP maps of the halogenated anesthetics: (**a**) Halothane; (**b**) Isoflurane; (**c**) Desflurane; (**d**) Sevoflurane.

Molecular electrostatic potential maps are very useful tools for obtaining information about the electron-rich and electron-deficient parts of a given molecule. MEP mappings for the molecules under study have been derived from CCSD computations employing the 6-311 + G(2d,p) basis set at an isovalue threshold of 0.0004, as depicted in Figure 3. It has been consistently noted that oxygen atoms tend to harbor negative charge distributions, while the hydrogen atoms bonded to oxygen predominantly constitute the most electropositive zones across the molecules. These distinct regions of charge polarity are instrumental in the establishment of non-covalent interactions, notably hydrogen bonding, which play a critical role in the interaction mechanisms observed between ligand and receptor in both molecular docking studies and molecular dynamics simulations.

Solvation occurs when the intermolecular forces between the solvent and solute particles are more significant than the intramolecular forces holding the solute particles together. Therefore, the stronger the intermolecular forces, the stronger the attraction. The solvent particles separate the solute particles, pull them apart, and surround them. The surrounded solute particles then move away from the remaining solute particles and enter the solution. The solvent and solute molecules reorganize into solvation complexes via bond formation, hydrogen bonding, and van der Waals forces. Polar molecules can easily solvate ions because they can position the partially charged portion of the molecule towards the ion owing to electrostatic attraction.

The solvation-free energy ΔG_solv_° plays an important role in computational chemistry because it can significantly contribute to the total free energy of chemical reactions in solution. Most practical calculations of ΔG_solv_° are based on the continuum solvation model. Usually, the computed ΔG_solv_° is represented by the sum of the electrostatic energy E_elst_ and the correction term ΔG_corr_°, which mainly describes non-electrostatic effects. The solvation energy is the energy associated with dissolving a solute in a solvent. It is positive if the dissolution process is endothermic and negative if it is exothermic. Halothane, isoflurane, desflurane, and sevoflurane have solvation energies of −0.112, isoflurane −0.127 kcal/mol, 0.137, and −0.142 kcal/mol, respectively.

**Figure 4:**
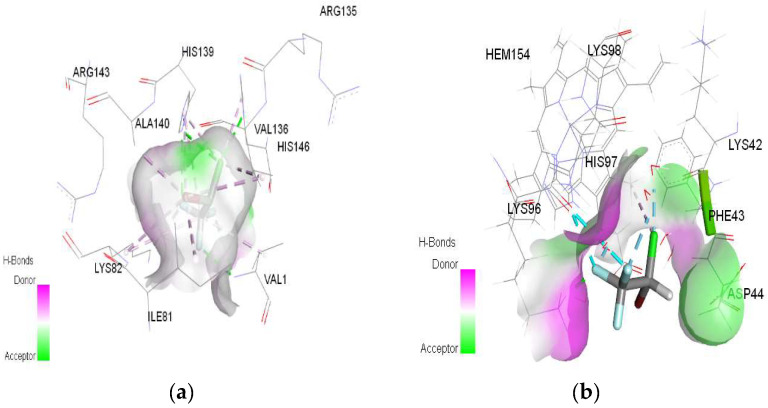
3D Target–ligand interaction map: (**a**) Halothane-HMG; (**b**) Halothane-MGB, at the end of 100 ns MD simulations.

**Figure 5:**
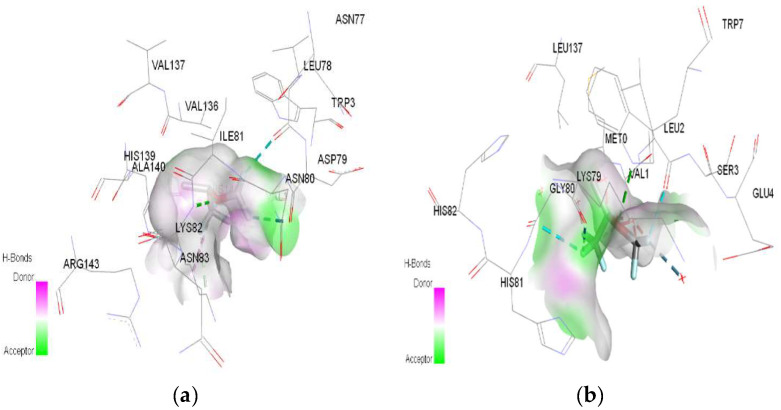
3D Target–ligand interaction map: (**a**) Isoflurane-HMG; (**b**) Isoflurane-MGB, at the end of 100 ns MD simulations.

**Figure 6:**
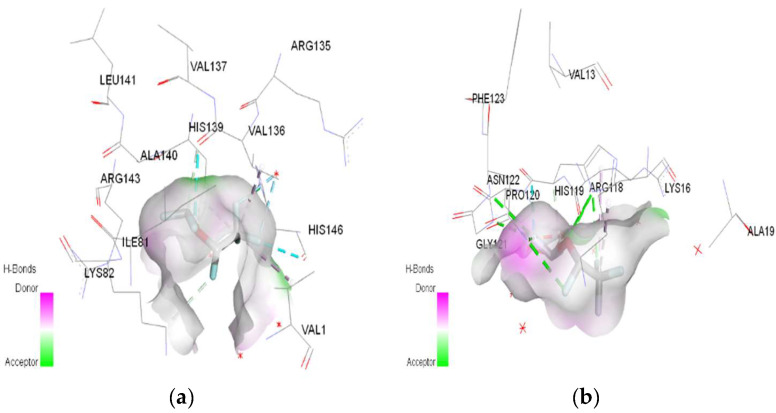
3D Target–ligand interaction map: (**a**) Desflurane-HMG; (**b**) Desflurane-MGB, at the end of 100 ns MD simulations.

**Figure 7:**
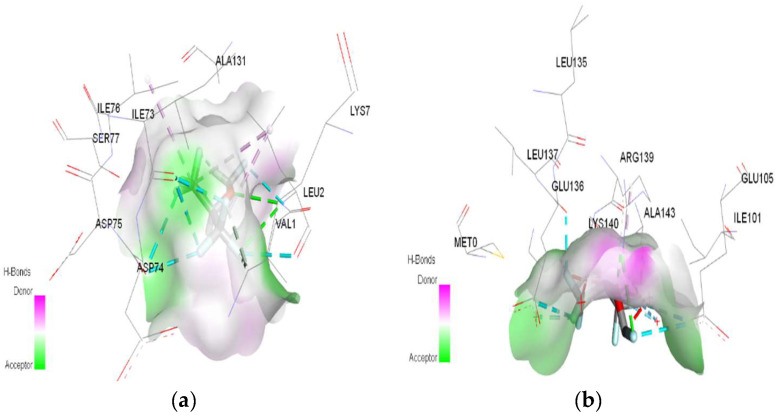
3D Target–ligand interaction map: (**a**) Sevoflurane-HMG; (**b**) Sevoflurane-MGB at the end of 100 ns MD simulations.

**Figure 8:**
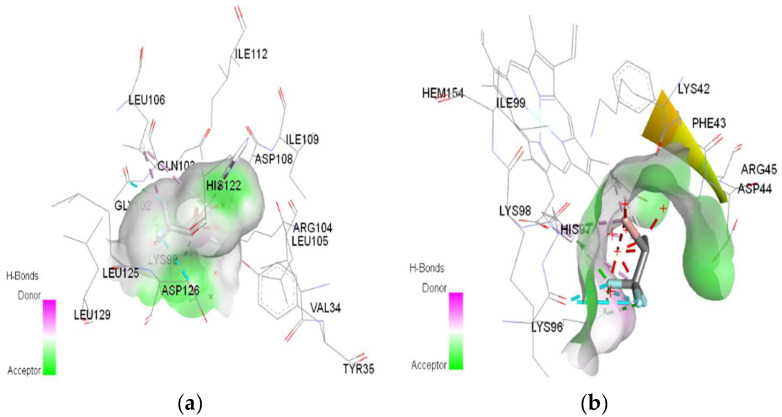
3D Target–ligand interaction map: (**a**) Halothane-HMG-HCl; (**b**) Halothane-MGB-HCl, at the end of 100 ns MD simulations.

**Figure 9:**
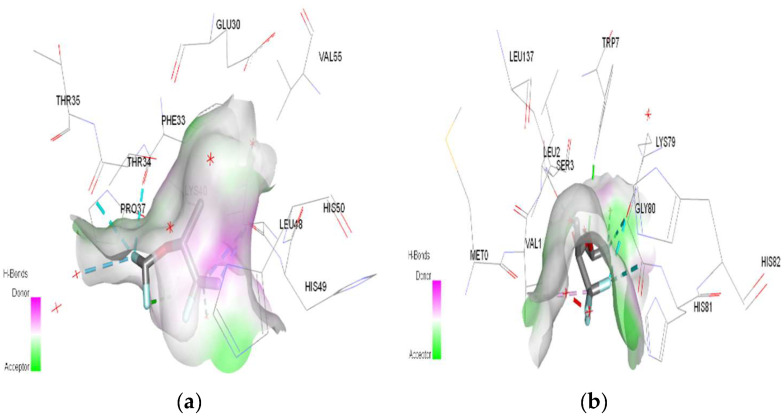
3D Target–ligand interaction map: (**a**) Isoflurane-HMG-HCl; (**b**) Isoflurane-MGB-HCl, at the end of 100 ns MD simulations.

**Figure 10:**
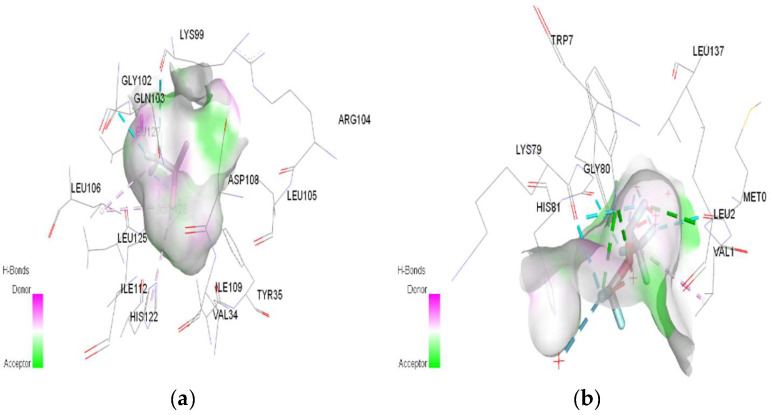
3D Target–ligand interaction map: (**a**) Desflurane-HMG-HCl; (**b**) Desflurane-MGB-HCl, at the end of 100 ns MD simulations.

**Figure 11:**
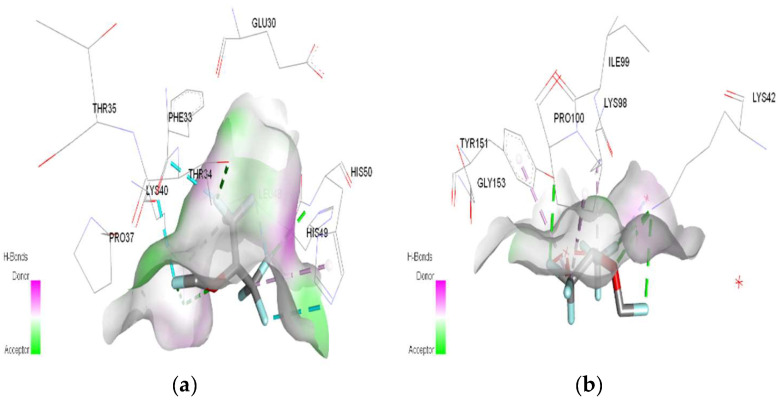
3D Target–ligand interaction map: (**a**) Sevoflurane-HMG-HCl; (**b**) Sevoflurane-MGB-HCl, at the end of 100 ns MD simulations.

In molecular docking calculations, 30 top-scoring docked poses have been obtained for each compound. The highest docking scores for each compound are given in Table 1, Table 2 and Table 3. After molecular docking calculations, each top-scoring ligand–receptor complex was subjected to 100 ns MD simulations to investigate the stability of ligand–receptor complexes and to investigate the ligand–receptor interactions in more detail. Three-dimensional maps of ligand–receptor interactions at the end of each 100 ns MD simulation are given in Figure 4, Figure 5, Figure 6, Figure 7, Figure 8, Figure 9, Figure 10 and Figure 11.

### 2.2. Ligand–Target Binding Energy Value of Hemoglobin–Hydrochloric Acid and Myoglobin–Hydrochloric Acid Complexes to Volatile Anesthetics

After Autodock 4.2.6 and AutoDock Vina redocking, RMSD (root-mean-square deviation) was calculated in both cases. The results were low RMSD values (all of them are ≤0.8 Å), suggesting that our preliminary docking methodology is robust.

The results showed that all ligands bind to the target and remain there throughout the MD simulations, remaining in the binding pocket until the end of the simulation.

The results also showed that mainly hydrogen bonds (classical and non-classical) and some other types of interactions [(pi–hydrophobic, alkyl hydrophobic, mixed pi/alkyl hydrophobic, and halogen bonds (fluorine)] participate in stabilizing the complexes.

**Figure 12:**
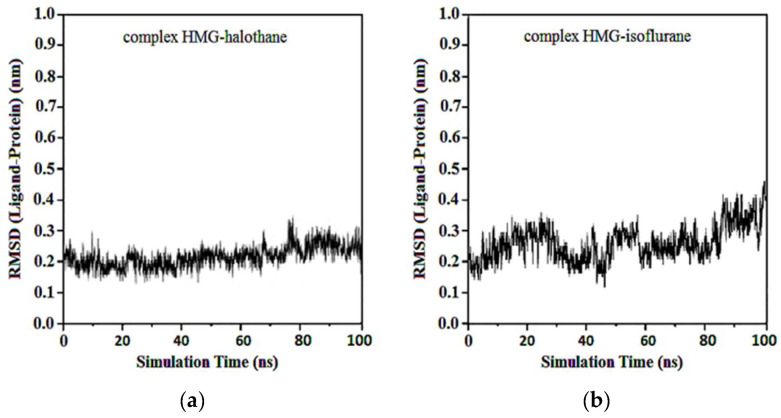

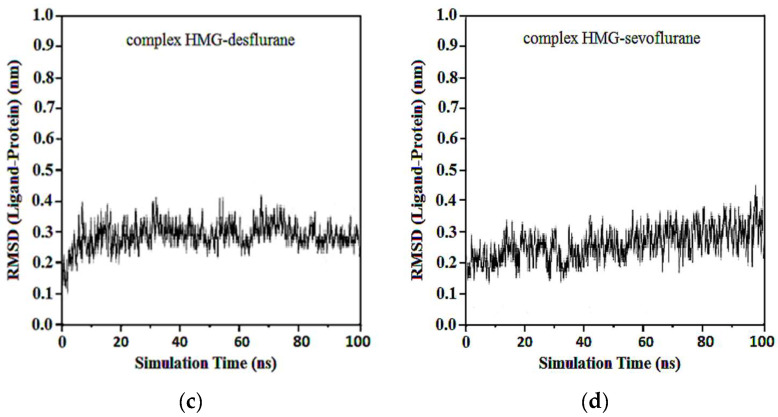
RMSD ligand-HMG: (**a**) HMG-Halothane; (**b**) HMG-Isoflurane; (**c**) HMG-Desflurane; (**d**) HMG-Sevoflurane.

The RMSD of ligand compared to the position of the target was monitored for each complex to investigate how well the binding pose was preserved during the MD simulation. The results showed that in the complexes of HMG and halothane, isoflurane, desflurane, and sevoflurane, the position of the ligands was preserved during the entire simulation. Average RMSDs and standard deviations were found to be 0.17 ± 0.03, 0.19 ± 0.05, 0.21 ± 0.02, and 0.22 ± 0.02 (Figure 12).

**Figure 13:**
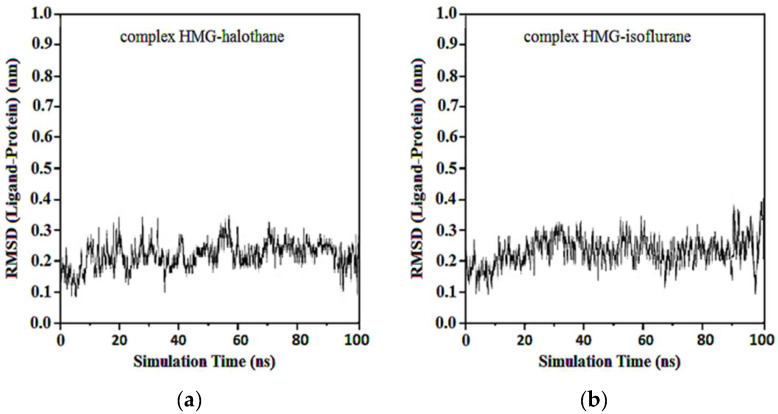

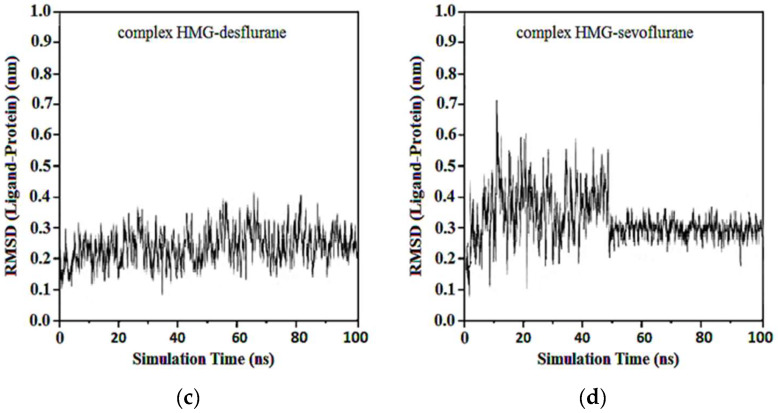
RMSD ligand-protonated HMG: (**a**) Protonated HMG-Halothane; (**b**) Protonated HMG-Isoflurane; (**c**) Protonated HMG-Desflurane; (**d**) Protonated HMG-Sevoflurane.

In the complexes of compounds between protonated HMG and halothane, isoflurane, desflurane, and sevoflurane, no considerable change in the position of the ligand was observed after the first 50 ns. Average RMSDs and standard deviations were found to be 0.17 ± 0.03, 0.19 ± 0.04, 0.22 ± 0.02, and 0.22 ± 0.03 nm for the complexes of protonated HMG-ligands. The results showed that in all ligand–receptor complexes, the HMG target remained stable throughout the MD simulation (Figure 13). For sevoflurane, the baseline change around 50 ns likely corresponds to a significant event in the molecular dynamics simulation of the ligand–target complex that indicates a transition phase where the ligand repositions within the binding site and shows a change in interaction dynamics such as the formation of key hydrogen bonds and hydrophobic interactions.

**Figure 14:**
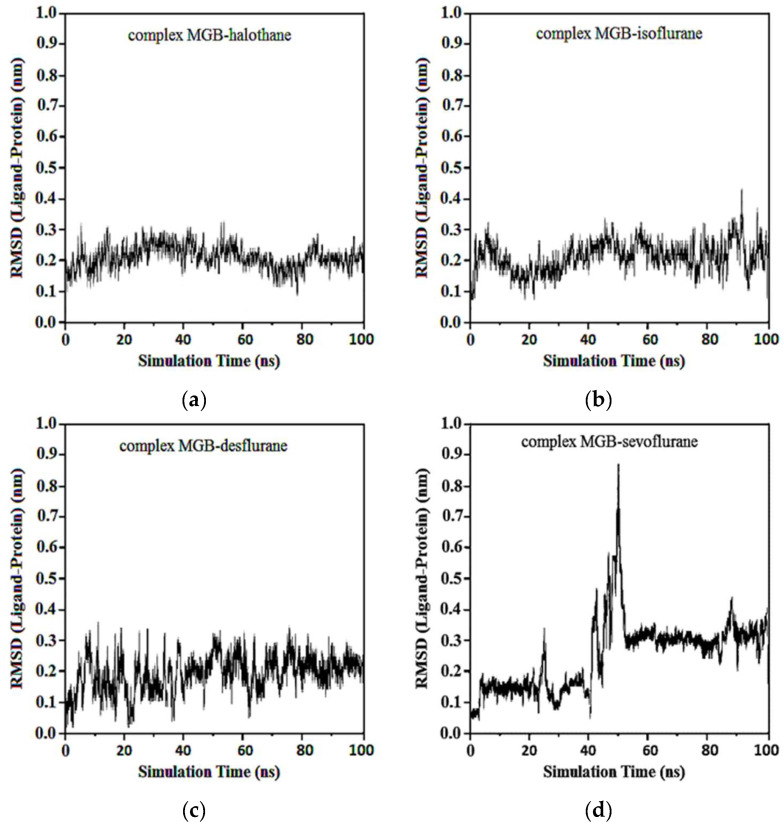
RMSD ligand-MGB: (**a**) MGB-Halothane; (**b**) MGB-Isoflurane; (**c**) MGB-Desflurane; (**d**) MGB-Sevoflurane.

Average RMSDs and standard deviations for MGB with halothane, isoflurane, desflurane, and sevoflurane were found to be 0.19 ± 0.02, 0.19 ± 0.02, 0.20 ± 0.04, and 0.23 ± 0.06. In the complexes of MGB-ligands compound, halothane, isoflurane, and desflurane quickly reached their equilibrium position and remained in this position during almost the entire simulation. Sevoflurane was observed to be detached from the MGB target at the 40th ns and reattached at the 50th ns, remaining in the binding pocket until the end of the simulation (Figure 14).

**Figure 15:**
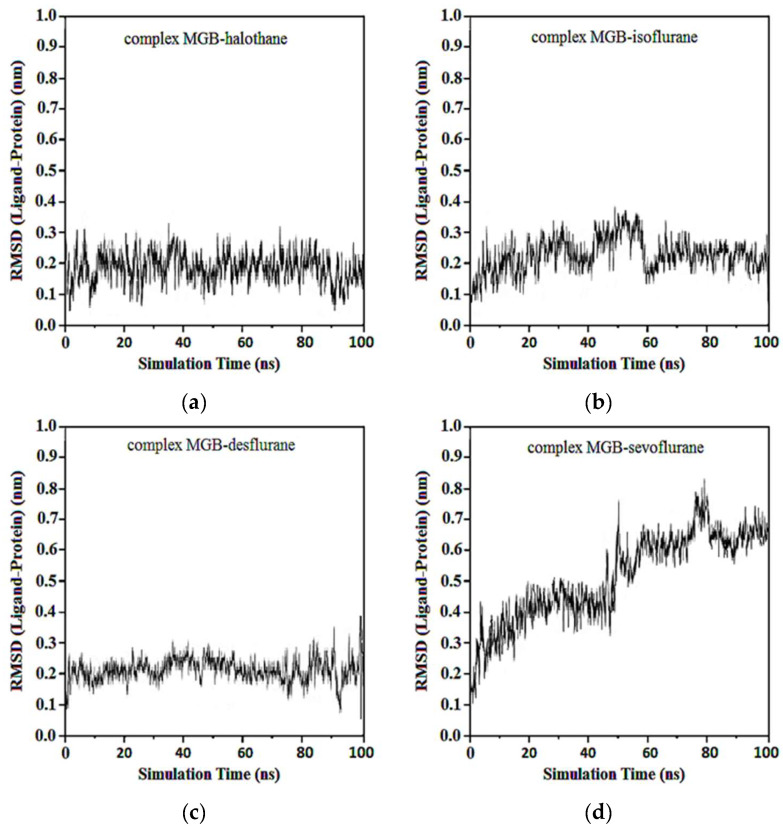
RMSD ligand-protonated MGB: (**a**) Protonated MGB-Halothane; (**b**) Protonated MGB-Isoflurane; (**c**) Protonated MGB-Desflurane; (**d**) Protonated MGB-Sevoflurane.

Average RMSD and standard deviations for protonated MGB with halothane, isoflurane, desflurane, and sevoflurane were found to be 0.23 ± 0.02, 0.23 ± 0.03, 2.22 ± 0.02, and 2.22 ± 0.05, respectively (Figure 15). Similar to Figure 13d, the baseline change around 50 ns in Figure 15d, the abrupt change at this specific time point, indicates alterations in the ligand’s binding dynamics and stability within the myoglobin binding site. This appears due to adjustments in the protein–ligand complex as it reaches a new equilibrium state and due to significant molecular interactions such as changes in salt hydrogen bonds or hydrophobic interactions crucial for stabilizing the ligand within the myoglobin binding site.

These observations indicate the complex nature of protein–ligand interactions and the sensitive balance of forces that govern them in a dynamic simulation environment. Each change in the simulation trajectory provides insights into the potential impacts of hydrochloric acid on the binding efficacy and stability of anesthetic molecules in fire smoke inhalation scenarios, crucial for understanding their pharmacodynamics and therapeutic implications.

The radius of gyration (R_G_) is another parameter that is used to investigate the stability of the protein. R_G_ of the enzyme was monitored during the MD simulation. Average R_G_s and standard deviations of the HMG target in the complexes with halothane, isoflurane, desflurane, and sevoflurane were found to be 2.23 ± 0.01, 2.21 ± 0.01, 2.22 ± 0.01, and 2.23 ± 0.01, respectively.

Average R_G_s and standard deviations of the protonated HMG target in the complexes with halothane, isoflurane, desflurane, and sevoflurane were found to be 2.22 ± 0.01, 2.23 ± 0.02, 2.22 ± 0.01, and 2.23 ± 0.01 nm, respectively. Additionally, average R_G_s and standard deviations for MGB with halothane, isoflurane, desflurane, and sevoflurane were found to be 2.22 ± 0.01, 2.22 ± 0.03, 2.21 ± 0.02, and 2.23 ± 0.02, respectively. Average R_G_s and standard deviations for protonated MGB with halothane, isoflurane, desflurane, and sevoflurane were found to be 2.20 ± 0.02, 2.21 ± 0.02, 2.22 ± 0.02, and 2.23 ± 0.03, respectively.

**Figure 16:**
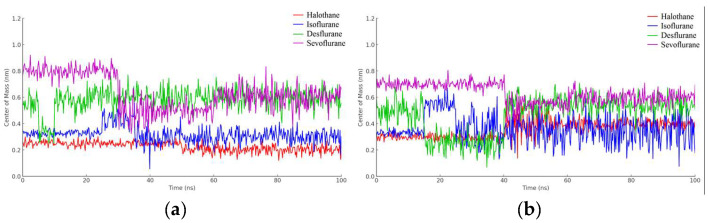
Distance fluctuation between the center of mass of HMG (residue VAL1) and ligands over 100 ns simulation time: (**a**) HMG; (**b**) Protonated HMG.

In Figure 16a, the red line corresponds to halothane, which displays the most constrained fluctuation profile, indicating a tighter and possibly more stable interaction with hemoglobin. Isoflurane (blue) and desflurane (green) exhibit intermediate levels of fluctuation, suggesting a more dynamic interaction with variable distances between the centers of mass. Sevoflurane (purple), in contrast, demonstrates the highest degree of fluctuation, indicating the most significant mobility and the least stable interaction within the hemoglobin binding site.

These results provide valuable insights into the relative binding dynamics of different anesthetic molecules with hemoglobin, which could affect their transport, affinity, and efficacy. Moreover, understanding these interactions at the molecular level could potentially inform the design of new anesthetics with optimized properties for clinical applications.

The halothane-protonated hemoglobin complex in Figure 16b exhibits the slightest distance fluctuation, suggesting the most stable interaction due to stronger binding affinities or favorable electrostatic interactions. Isoflurane and desflurane show intermediate fluctuations, indicating a balance between binding affinity and conformational flexibility. Sevoflurane’s trace is characterized by the greatest variability, implying a more dynamic interaction and potentially weaker or more transient binding events.

**Figure 17:**
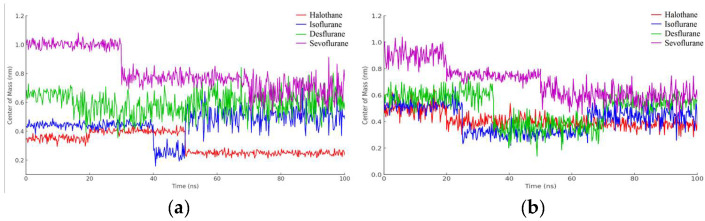
Distance fluctuation between the center of mass of MBG (residue ILE111) and ligands over 100 ns simulation time: (**a**) MGB; (**b**) Protonated MGB.

In Figure 17a, observations from the trajectories reveal that halothane exhibits minimal fluctuation, implying a more consistent and potentially stronger interaction with myoglobin. Isoflurane and desflurane present with moderate levels of fluctuation, suggesting a dynamic interplay between stable binding and mobility. Sevoflurane shows the highest fluctuation amplitude, hinting at the most dynamic interaction and possibly weaker or less consistent binding interactions.

Lastly, the graphical representation from Figure 17b for halothane (red) shows the lowest amplitude of fluctuation, suggesting a relatively stable interaction with protonated myoglobin. Isoflurane (blue) and desflurane (green) display intermediate fluctuation amplitudes, indicating a mix of stable and dynamic interactions, while Sevoflurane (purple) exhibits the highest amplitude of fluctuations, possibly reflecting less stable binding dynamics and higher mobility within the binding pocket of the protein.

## 3. Discussion

Mass fire accidents frequently occur around the world, and the consequent inhalation of combustion gases represents the leading cause of death under these circumstances. Typically, these gases consist, among others, of carbon dioxide (CO_2_), water (H_2_O), carbon monoxide (CO), hydrogen cyanide (HCN), hydrochloric acid (HCl), nitrogen oxide (NO_x_), and sulfur oxide (SO_x_).

The replication of major events, including the spread and maintenance of fire, temperature and pressure, and toxic atmosphere, cannot be achieved even in high-performance laboratories and simulators. As a result, conducting typical clinical trials in emergency departments or pre-hospital settings for mass burns incidents is challenging. Furthermore, retrospective observational studies usually fail to include a significant number of patients with similar pathology profiles and ages under ethically binding conditions. This makes it challenging to draw scientifically relevant conclusions from in vivo studies, especially in cases of catastrophic events [33].

By incorporating current molecular research methodologies and computational chemistry-specific models and techniques, we can gain real-time insights into the intricate workings of smoke poisoning caused by fire without relying on living organisms [34]. This approach can potentially provide valuable information that can aid in our understanding of the biological mechanisms underlying smoke poisoning. Data predicted by theoretical models can be used in practical studies, helping to identify the relationship between specific changes in conformational or energetic properties and therapeutic effects, side effects, and associated risks of using pharmacological substances in medical practice, especially in special emergency circumstances.

Currently, there are protocols to address different types of emergencies, including poisoning and burns [35]. Despite the growing concern around smoke poisoning, there is still no agreement on its specific treatment methods, in particular, regarding the most indicated anesthetic protocols for smoke-intoxicated patients. Additionally, there is a lack of standardization in integrated prehospital and emergency departments when it comes to managing fire smoke poisoning, in particular, generated by chlorine inhalation, regardless of whether the incident occurred in a civil, industrial, domestic, or deliberate setting. This gap in knowledge and practice requires urgent attention and research to better support the care of patients affected by smoke poisoning and burns in mass burning incidents [36,37,38,39].

The fact that myoglobin is mainly blocked and unavailable during smoke poisoning is an important aspect that may influence the therapeutic approach and risk stratification through muscle and myocardial damage severity. The findings on the affinity of hydrochloric acid for both myoglobin and hemoglobin highlight its irritating effects and cellular toxicity, particularly on myoglobin and oxygen transport (Table 1).

In emergency cases where the chemical determination by laboratory analysis is not widely available or does not provide the necessary information for a better prognosis, computational modeling results can be used as tactical reasoning tools for re-standardizing smoke poisoning management. This can be particularly helpful for substances that are not easily identifiable through emergency laboratory analysis and when quick decisions need to be made to improve patient outcomes [40].

In our theoretical study, we observed that the association of protons (H^+^ ions) with the amino acids in hemoglobin/myoglobin causes a conformational change in protein folding, ultimately reducing the affinity of the binding sites for oxygen molecules (Table 1).

The molecular docking analysis reveals changes in the binding site and binding energy of certain gaseous anesthetics. The analysis also shows that hemoglobin forms more stable complexes with gaseous anesthetics than myoglobin (Table 2). Moreover, the protonation of the targets with hydrochloric acid does not significantly influence the binding energy of gaseous anesthetics at their level (Table 3).

Different types of non-covalent interactions, such as classic hydrogen bonds, hydrophobic interactions, halogen bonds, miscellaneous and unfavorable bonds, are established between gaseous anesthetics and targets.

In the case of halothane, the protonation of the HMG and MGB targets does not change the ligand–target interactions (Table 3, Figure 8). Halothane interacts with HMG through two pi–alkyl bonds (LEU C:106 and HIS C:122). With the amino acids ASP C:126, GLY C:102, and LYS C:99, it forms three halogen bonds (fluorine). Halothane interacts with MGB through two classical hydrogen bonds (LYS A:96, HIS A:97) and two pi–alkyl interactions with HEM (A:154). Also, at the level of the active site, hydrogen bonds are formed between halothane and water molecules (HOH A:305).

In the case of isoflurane, the binding site at the level of hemoglobin and myoglobin remains unchanged, with protonation of the target not changing the binding mode at the level of the active site (Table 3). Between isoflurane and HMG, a classical hydrogen bond is formed (GLN C:103), two non-classical hydrogen interactions (TYR D:35, ASP C:126), four halogen bonds (fluorines) with LYS C:99, GLY C:102, HIS C:122, an alkyl interaction with LEU C:129. A classic hydrogen bond (TRP A:7), four halogen bonds (fluorines) were formed between isoflurane and MGB, involving LEU A:2, LYS A:79, GLY A:80; a hydrophobic alkyl interaction with VAL A:1.

The water molecules in the active sites of HMG and MGB form hydrogen bonds with the ligands but also unfavorable bumps (Table 3). Unfavorable bonds affect the stability of the ligand–target complex. The formation of any unfavorable bond between/in the target–ligand complex reduces the stability of the complex as these types of bonds indicate a force of repulsion occurring between molecules/atoms.

In the case of desflurane, the protonation of the HMG target partially changes the binding mode, leaving some common amino acids in the binding site of the normal or protonated target (VAL B:1, LYS B:82, HIS B:139). In the case of MGB, protonation radically changes the binding mode of desflurane to the target (Figure 10). Desflurane interacts with normal MGB through three classic hydrogen bonds with the amino acids HIS A:119, GLY A:121, ASN A:122; p interaction pi–alkyl u LYS A:16, a halogen (fluorine) bond with ARG A:118. With protonated MGB, desflurane forms two classic hydrogen bonds (LEU A:2, TRP A:7), four halogen bonds (fluorines) with LYS A:79, GLY A:80, MET A:10, an alkyl interaction (WAVE A:1).

In the case of sevoflurane, the ligand–target binding sites change radically after the protonation of HMG and MGB, respectively (Figure 11).

Throughout the MD simulations, ligands generally maintained their positions in the binding pockets of the target proteins, indicating stable complexes. Sevoflurane was an exception, as it detached and reattached after ten ns but remained in the binding pocket until the end of the simulation. The negative and positive charge centers observed in the MEP maps contributed to the formation of non-bonded interactions, particularly hydrogen bonds, which were instrumental in the stability of the ligand–receptor complexes during molecular docking and MD simulations.

R_G_ was used to investigate the stability of the protein during the MD simulation. No considerable change was observed in the R_G_ of the target protein, indicating stability throughout the simulation. Average R_G_s for the HMG target in complexes with halothane, isoflurane, desflurane, and sevoflurane were found to be consistently around 2.22 ± 0.01 nm, demonstrating the structural stability of the protein in these simulations.

Our study, which used theoretical chemistry methods and calculation, found that the ability of hemoglobin and myoglobin to bind and transport oxygen is influenced by protonation in the hydrochloric acid-rich environment of fire smoke. Furthermore, the use of halogenated anesthetics on patients exposed to fire smoke with a high concentration of hydrochloric acid can cause a further reduction in the binding affinity of oxygen. This effect is more pronounced in hemoglobin and is greater when desflurane and sevoflurane are used due to conformation changes in the pathways, sites, and binding energies (Table 3).

Other laboratory studies confirmed that halogenated ethers, especially isoflurane, desflurane, and sevoflurane, can shift the oxyhemoglobin dissociation curve (ODC) to the left or right, thus increasing or decreasing the hemoglobin–oxygen affinity [41]. With increasing concentrations from control to medium, desflurane and isoflurane significantly decreased HMG-O_2_ affinity by shifting the ODC to the right, but sevoflurane showed no effects. When concentrations were further increased from medium to high, all three volatile anesthetics shifted the ODC back to the left. Comparing only controls to high concentrations, a significant increase in HMG-O_2_ affinity for desflurane and sevoflurane was detected. These effects of anesthetic–(myoglobin/hemoglobin)–hydrochloric acid interactions become sensitive in clinical practice on patients with smoke poisoning, adding to the non-specific effects of halogenated agents on the cardiovascular and respiratory system [42,43,44,45,46,47,48,49,50,51]. Numerous researchers have focused their studies on identifying the specific differences in the pharmacological and physicochemical characteristics of the main representatives most used in the category of halogenated anesthetics. They found that volatile anesthetic agents provide cardioprotective benefits by reducing or preventing myocardial ischemia both intraoperatively and postoperatively, maintaining the left ventricular function, decreasing stress-induced cell death, and maintaining myocyte mitochondrial function [52,53,54]. These agents generate a myocardial preconditioning mechanism similar to ischemic preconditioning using the same cellular pathways [55,56,57,58,59,60,61,62]. Among them, halothane causes the most significant reduction in the cardiac index, while sevoflurane, desflurane, and isoflurane have a lesser effect. Halothane mainly lowers blood pressure by decreasing myocardial contractility, while modern volatiles primarily affect systemic vascular resistance [63]. However, in situations where the presence of desflurane and sevoflurane modifies the stability and binding affinity of hemoglobin and myoglobin with oxygen, isoflurane remains the anesthetic agent with the most significant cardioprotective effect. This is especially important when considering primary myocardial injury related to myoglobin blockade and secondary injury related to the reduction in oxygen affinity of hemoglobin.

Last but not least, it is essential to consider that the exact action mechanism of volatile anesthetics is still unknown. There are several hypotheses, but the most widely accepted one is the Meyer–Overton theory of fat solubility [64]. This hypothesis is based on two observations: first, the potency of inhalation anesthetics is highly correlated with their solubility in fats, and second, their structure is very diverse [65]. This theory suggests that halogenated agents most likely affect the lipid layer of cell membranes. More recent approaches have explored the binding of halogenates to protein membrane receptors, revealing that they act on gamma-aminobutyric acid (GABA) type A and glycine receptors, which facilitate chlorine conductance [66].

The framework suggests that the chlorine ion may be the crucial point in the model of halogenated anesthetics interaction in smoke-intoxicated patients. However, many aspects related to the pharmacokinetics of these anesthetics remain under discussion in this specific context. Several other factors affect the rate at which an anesthetic reaches its target concentration in the brain: pressure differences at the level of the alveolar–capillary membrane; the degree of solubility of the agent in the blood (the effect on the target organ is dependent on the partial pressure of the volatile agent and inversely proportional to its solubility); patient-related factors such as cardiac output or the ventilation/perfusion ratio; the inspired fraction of the halogenate.

The study has two significant limitations.

One concern relates to using a single pH value, specifically pH = 7.00, when calculating the protonation of the target. Although this choice is realistic and has a pathophysiological basis, different hypotheses suggest changes in protonation characteristics at different pH levels. Therefore, it may be necessary to investigate the comparative outcomes obtained with a broader pH range, beginning with severe acidosis (pH = 6.8) and up to moderate acidosis (pH = 7.15). This would be the field that consistently requires therapeutic intervention and, on the other hand, corresponds to a pathological condition compatible with the need for general anesthesia, and in which the various adverse effects of anesthetics can have major clinical significance and impact.

A second momentary limitation of the study is represented by the fact that we have not identified in the literature experimental studies using animal models aimed at identifying the different actions of halogenated anesthetics in subjects who were exposed to the inhalation of hydrochloric acid or significant variations of pH.

These types of experimental studies can offer the possibility to verify in practice how the conformational and stability changes of the molecules resulting from the studied interactions correlate with changes in pharmacological action (dynamics or kinetics).

As the different structure of these molecules determines particularities of the anesthetic or secondary effects, it is expected that the structural changes generated by the post-inhalation interactions of fire smoke rich in hydrochloric acid, predicted by computational methods, will have an impact on the specific action, in any of the directions of interest: anesthetic effect, myocardial depression, atrioventricular conduction, bronchodilation, irritant effect on the upper airway. Either of these changes may become susceptible to bringing new arguments in the specific protocol for the use of one or another of these inhalational anesthetics in certain smoke-intoxicated patients who present certain specific morbid conditions or particular pathological antecedents.

Currently, it is still unclear which of the structural aspects (bonds, configurations) of each halogenated anesthetic molecule determines each therapeutic action or adverse effect; computational chemistry can only predict the interaction patterns between targets and ligands without being able to specify or anticipate clinical–therapeutic correlations.

While the underlying intimate mechanisms of interaction remain unclear, not only the minimum alveolar concentration (MAC) of anesthetics but also the pH, temperature, and partial concentration of hydrochloric acid could generate clinically significant effects that are not available at this moment. These results show an impact of interaction that needs to be further investigated to determine if patients undergoing anesthesia may potentially benefit or become disadvantaged from this slight increase or decrease in HMG/MGB-O_2_ affinity or different anesthetic effects.

Further research on the interaction between halogenated anesthetics, myoglobin, and hemoglobin in fire smoke-intoxicated patients is necessary. It must focus on developing experimental models that can highlight and associate clinical evidence with the theoretical chemistry component. This association promises important steps toward developing safe, personalized, and optimal anesthesia protocols.

The ideal anesthetic agent for these patients needs to offer certain advantages. It should not significantly affect oxygen transport to the tissues, it should have broncho-dilator and cardioprotective effects, and it should not cause laryngospasm. Additionally, it must be easily accessible in emergency departments and not require extensive and complex monitoring to determine bioavailability. Halogenated anesthetics meet many of these requirements.

## 4. Materials and Methods

The different features of electrostatics on protein–protein interactions are already known and have been demonstrated by numerous authors, who showed that electrostatic interactions are the most important force for the stability, binding profile, and function of proteins [67]. Electrostatics is the driving force behind electron transfer and protein–protein association [68]. The role of electrostatics in protein binding energy and its dependence on different conditions has been outlined in a series of studies [69,70,71].

Multiple studies have aimed to appreciate the possible protonation changes associated with binding interactions [72,73]. Some authors have raised the issue of the importance of protonation reactions in receptor–ligand recognition [74]. There is little experimental evidence that protonation states may not be constant in ligand–target complexes but may change during the reaction. Based on this experimental evidence of target–ligand interactions, it seems evident that such data are not absolute and can be dependent on the external conditions of the experiments, such as the pH, temperature, concentration, or concentration of other molecules [75].

In our study, quantum chemical calculations were performed using the Gaussian program suite (Semichem Inc., Shawnee Mission, KS, USA) at coupled cluster single-double and perturbative triple [CCSD(T)]/6-311 + G (2d,p) optimization to improve the ligands molecular geometry. Applying these calculations to the myoglobin and hemoglobin models, we were able to study their binding to the essential components of hydrochloric acid toxic fire smoke [76]. The purpose of in silico testing is to observe the influence of hydrochloric acid on myoglobin/hemoglobin targets in the mode/energy of gaseous anesthetic target binding.

Different molecules differ in shape, functional groups, surface, and ability to form hydrogen bonds and, therefore, will experience different consequences due to the interactions of all these factors. Only in very few cases do experimental data exist for the protonation states before and after binding. In most cases, these protonation states (and related changes in reactions) must be predicted using theoretical chemistry software (https://playmolecule.org/proteinPrepare, accessed on 23 June 2023). However, another frequently unknown factor is the pH of binding. The binding may not involve protonation changes at certain pH values, whereas at other pH levels, it may involve changes [71,72].

Therefore, computing the binding free energy or predicting the binding mode via ab initio docking while considering protonation and conformational changes in the entire domain at physiological pH is still challenging [77].

Considering and analyzing all these arguments, we achieved protonation of the target at pH = 7.00 [78]. We considered that under conditions of significant hypoxic aggression, such as that by smoke inhalation, especially with hydrochloric acid in its composition, hyperchloremic metabolic acidosis is highly likely [79]. At the same time, significant acidosis is frequently found in a patient whose general condition requires general anesthesia, outlining more precisely and more realistically the framework for verifying the theory postulated by us.

### 4.1. Ligands Preparation and Target Preparation

The three-dimensional coordinates of all ligands were generated using the Gaussian program suite by applying the CCSD(T)/6-311 + G (2d,p) level of theory. While the theoretical differences between the enantiomers of chiral anesthetic agents like halothane, desflurane, and isoflurane exist, they are not significant in terms of their clinical application, with all being used as racemic mixtures, so in the docking and molecular dynamic calculations we used the S enantiomers.

Solvation calculations were performed using a C-PCM with water as the solvent. Mulliken population analysis has also been used to understand the reactive parts of molecules.

The X-ray crystal structures of the targets were retrieved as target.pdb files from the major protein databases, Protein Data Bank [80], and optimized with ModRefiner software (https://zhanggroup.org/ModRefiner/, accessed on 20 July 2023) [81]. The target codes were 3WTG code (resolution 2.3 A) and myoglobin (2SPL code, resolution 1.7 A). The targets were prepared by adding all the polar hydrogens, maintaining the water molecules, and computing the Gasteiger charge.

### 4.2. Molecular Docking and Molecular Dynamics Simulation Studies

AutoDock is a molecular docking program that is widely used in academic research. It is based on a semi-empirical free energy force field and offers a variety of search algorithms, including a Monte Carlo Simulated Annealing algorithm, a Genetic Algorithm (GA), and a Lamarckian Generic Algorithm (LGA), which is a rapid hybrid local search GA. One of the key features of the semi-empirical free energy force field is the use of an advanced thermodynamic model to simulate the ligand–target binding process. Additionally, it includes a full desolvation model and terms for all the atom types.

The binding energy of the compound was calculated by using the following formula:

Binding energy = A + B + C − D

where A denotes final intermolecular energy + van der Waals energy (vdW) + hydrogen bonds + desolvation energy + electrostatic energy (kcal/mol), B denotes final total internal energy (kcal/mol), C denotes torsional free energy (kcal/mol) and D denotes the unbound system’s energy (kcal/mol).

We perform the molecular docking analysis using Autodock 4.2.6 with the molecular viewer and graphical support AutoDockTools [82].

In the docking protocol, for the protein targets, we create the grid box using Autogrid 4 with 120 Å × 120 Å × 120 Å in x, y, and z directions and 0.5 Å spacing from the target molecule’s center.

For the docking process, we chose the Lamarckian Genetic Algorithm (genetic algorithm combined with a local search), with a population size of 150, a maximum of 2.5 × 1062.5 × 106 energy evaluations, a gene mutation rate of 0.02, and 50 runs. We adopted default settings for the other docking parameters and performed all calculations under vacuum conditions. All the results from AutoDock were exported to PyMOL [83] and the Discovery Studio (Biovia) molecular visualization system [84].

Discovery Studio by BIOVIA provides a sophisticated suite of tools for the analysis and visualization of molecular interactions, which are crucial for understanding the structure, function, and dynamics of biomolecules in drug discovery and other areas of molecular biology. Below is a detailed discussion of the computational methods and algorithms employed by Discovery Studio for evaluating several key types of molecular interactions: conventional hydrogen bonds, unfavorable bumps (steric clashes), water-mediated hydrogen bonds, hydrogen–fluorine interactions, alkyl, and pi–alkyl interactions. This detailed account is suitable for an academic publication, providing insights into the intricacies of each interaction type analyzed.

In the case of conventional hydrogen bonds, the distance is less than 3.4 Å between the hydrogen donor and the acceptor atom. The angle formed by the donor atom, hydrogen atom, and acceptor atom should generally be between 90 and 180 degrees to be considered a valid hydrogen bond. This geometric criterion ensures that the interaction is energetically favorable and geometrically feasible, reflecting the directional nature of hydrogen bonds which are crucial for the stability and function of biological molecules.

Unfavorable bumps (steric clashes) are measured as the distance between the centers of any two non-bonded atoms. A clash is typically indicated when this distance is less than the sum of their van der Waals radii minus a tolerance factor (0.7 Å). This approach helps identify regions of potential structural strain or conflict in molecular models, highlighting areas that may require further optimization or indicate possible errors in structure prediction or docking simulations.

For water-mediated hydrogen bonds, the distance and angle criteria are similar to those of conventional hydrogen bonds. However, each hydrogen bond must separately meet these criteria involving a water molecule acting as a bridge between the donor and acceptor. This type of analysis is vital for understanding hydration shells around biomolecules, the role of solvents in enzyme activity, and the stability of ligand–protein complexes in aqueous environments.

For hydrogen (fluorine) interactions, the optimal distance for a hydrogen (fluorine) interaction is slightly longer (3.7 A) than that for a conventional hydrogen bond, acknowledging the smaller size and different electronic properties of fluorine. The angle should be in the range of 120–180 degrees, reflecting the strong directionality of these interactions. These interactions are of particular interest in medicinal chemistry, where fluorine is often used to modulate the activity and properties of pharmaceutical compounds.

Alkyl interactions evaluate the proximity of carbon atoms within alkyl groups, considering optimal distances that favor van der Waals interactions but do not necessarily adhere to strict bonding criteria (maximum 5.5 A). Alkyl interactions contribute to the hydrophobic core of proteins and the binding affinity in drug-target interactions, influencing molecular conformation and stability.

Pi–alkyl interactions are characterized by the proximity of an alkyl group to the face or edge of an aromatic ring (maximum 5.5 A). Effective interactions generally occur when the alkyl group is oriented edge to face or offset from the center of the pi system. Pi–alkyl interactions enhance the specificity and stability of molecular interactions in biological systems, playing a crucial role in molecular recognition and the structural integrity of complex biomolecules.

The performance of Autodock 4.2.6 was evaluated by redocking and then expressing the results as root-mean-square deviation (RMSD) in Å. All calculations were performed in duplicate, and the results are expressed as averages. The redocking involves overlapping the ligands to calculate the RMSD using Discovery Studio Visualization, Version 4.5 (Biovia) Discovery Studio software. We also ran a comparative RMSD analysis between Autodock 4.2.6 and AutoDock Vina to assess the docking method’s repeatability and reproducibility.

Thirty docking poses were obtained for each molecular docking calculation. After molecular docking calculations, the top-scoring ligand–receptor complexes were subjected to 100 ns all-atom MD simulations to investigate the ligand–receptor interactions in more detail and to determine the binding free energies accurately. In MD simulations, the GROMACS 5.1.4 program package was used [85]. The topology of the target was prepared using an AMBER (assisted model building with energy refinement) force field and TIP3P (transferable intermolecular potential with 3 points) water model. Ligand topologies were obtained from the SWISSPARAM Server; the tool used the MMFF94 force field to assign partial atomic charges to the molecular ligand structures. After neutralization of the system, energy minimization was performed for each complex by employing the steepest descent minimization algorithm with 5000 steps. After performing 200 ps NVT and NPT ensemble equilibrations, MD simulations were performed in a dodecahedron simulation box for 100 ns at 1 bar and 300 K reference pressure and temperature.

## 5. Conclusions

According to our theoretical model, the ability of hemoglobin and myoglobin to bind and transport oxygen is influenced by protonation in the hydrochloric acid-rich environment of fire smoke. Furthermore, the use of halogenated anesthetics on patients exposed to fire smoke with a high concentration of hydrochloric acid can cause multiple interactions. This effect is more pronounced in hemoglobin and is greater when desflurane and sevoflurane are used due to conformation changes in the pathways, sites, and binding energies.

The results show that halothane and isoflurane undergo no or minor conformational changes that affect the stability of the complex. In the case of desflurane, protonation of the hemoglobin target partially alters it, but for myoglobin, protonation radically changes the mode of binding to the target. Among the four anesthetic agents, sevoflurane undergoes a radical structural change in modifying the ligand–target binding sites, its anesthetic effect being the most unpredictable under these circumstances.

As patients from mass fire incidents are often in critical condition and require perfectly adapted and long-term anesthesia, further clinical studies are needed to confirm whether the changes that occur under these conditions lead to changes in the specific effects of these anesthetics when used in fire smoke-intoxicated patients.

## Figures and Tables

**Table 1:** The value of the ligand–target binding energy [kcal/mol].

Ligand	HMG	MGB
Hydrochloric acid	−2.33	−2.31

**Table 2:** The value of the ligand–target binding energy [kcal/mol].

Ligand	HMG	MGB
Halothane	−2.54	−2.07
Isoflurane	−1.88	−1.68
Desflurane	−1.58	−1.11
Sevoflurane	−1.46	−0.74

**Table 3:** The value of the ligand–target binding energy [kcal/mol].

Ligand	HMG-HCl	MGB-HCl
Halothane	−2.33	−2.09
Isoflurane	−1.61	−1.67
Desflurane	−1.36	−1.17
Sevoflurane	−1.15	−0.76

## Data Availability

Data are contained within the article.

## References

[1] Mat Kiah M.H. (2020). Toxic Species and Particulate Emissions from Synthetic Polymer Fires. Ph.D. Thesis.

[2] Doley P.M., Yuen A.C.Y., Kabir I., Liu L., Wang C., Chen T.B.Y., Yeoh G.H. (2022). Thermal Hazard and Smoke Toxicity Assessment of Building Polymers Incorporating TGA and FTIR—Integrated Cone Calorimeter Arrangement. Fire.

[3] Labadie M., Capaldo L., Courtois A., Mégarbane B. (2016). Mécanismes de Toxicité des Fumées d’incendie (monoxyde de carbone et cyanures exclus). Méd. Intensive Réanim..

[4] Borucka M., Mizera K., Przybysz J., Kozikowski P., Gajek A. (2023). Analysis of Flammability and Smoke Emission of Plastic Materials Used in Construction and Transport. Materials.

[5] Marongiu A., Faravelli T., Bozzano G., Dente M., Ranzi E. (2003). Thermal degradation of poly(vinyl chloride). J. Anal. Appl. Pyrolysis.

[6] Morim A., Guldner G.T. (2023). Chlorine Gas Toxicity.

[7] Anseeuw K., Delvau N., Burillo-Putze G., De Iaco F., Geldner G., Holmström P., Lambert Y., Sabbe M. (2013). Cyanide Poisoning by Fire Smoke Inhalation: A European Expert Consensus. Eur. J. Med..

[8] Rose J.J., Wang L., Xu Q. (2017). Carbon Monoxide Poisoning: Pathogenesis, Management, and Future Directions of Therapy. Am. J. Respir. Crit. Care Med..

[9] Ferrari L.A., Giannuzzi L. (2015). Assessment of Carboxyhemoglobin, Hydrogen Cyanide and Methemoglobin in Fire Victims: A Novel Approach. Forensic Sci. Int..

[10] Kinoshita H., Türkan H., Vucinic S., Naqvi S., Bedair R., Rezaee R., Tsatsakis A. (2020). Carbon Monoxide Poisoning. Toxicol. Rep..

[11] Antonio A.C., Castro P.S., Freire L.O. (2013). Smoke inhalation injury during enclosed-space fires: An update. J. Bras. Pneumol..

[12] Henry C.R., Satran D., Lindgren B., Adkinson C., Nicholson C.I., Henry T.D. (2006). Myocardial injury and long-term mortality following moderate to severe carbon monoxide poisoning. JAMA.

[13] Stapelberg F. (2020). Challenges in anaesthesia and pain management for burn injuries. Anaesth. Intensive Care.

[14] Bittner E.A., Shank E., Woodson L., Jeevendra Martyn J.A. (2015). Acute and Perioperative Care of the Burn-injured Patient. Anesthesiology.

[15] Bishop S., Maguire S. (2012). Anaesthesia and intensive care for major burns. Contin. Educ. Anaesth. Crit. Care Pain.

[16] Brusselaers N., Monstrey S., Vogelaers D., Hoste E., Blot S. (2010). Severe burn injury in Europe: A systematic review of the incidence, etiology, morbidity, and mortality. Crit. Care.

[17] Yassen K.A., Jabaudon M., Alsultan H.A., Almousa H., Shahwar D.I., Alhejji F.Y., Aljaziri Z.Y. (2023). Inhaled Sedation with Volatile Anesthetics for Mechanically Ventilated Patients in Intensive Care Units: A Narrative Review. J. Clin. Med..

[18] Joo H.S., Perks W.J. (2000). Sevoflurane Versus Propofol for Anesthetic Induction: A Meta-Analysis. Anesth. Analg..

[19] Chidambaran V., Costandi A., D’Mello A. (2015). Propofol: A review of its role in pediatric anesthesia and sedation. CNS Drugs.

[20] Jeleazcov C., Ihmsen H., Schmidt J., Ammon C., Schwilden H., Schuttler J., Fechner J. (2008). Pharmacodynamic modelling of the bispectral index response to propofol-based anaesthesia during general surgery in children. Br. J. Anaesth..

[21] Rigouzzo A., Girault L., Louvet N., Servin F., De-Smet T., Piat V., Seeman R., Murat I., Constant I. (2008). The relationship between bispectral index and propofol during target-controlled infusion anesthesia: A comparative study between children and young adults. Anesth. Analgesia.

[22] Sadhasivam S., Ganesh A., Robison A., Kaye R., Watcha M.F. (2006). Validation of the bispectral index monitor for measuring the depth of sedation in children. Anesth. Analgesia.

[23] Dahan A., Nieuwenhuijs D.J., Olofsen E. (2003). Influence of propofol on the control of breathing. Adv. Exp. Med. Biol..

[24] Kashiwagi M., Okada Y., Kuwana S., Sakuraba S., Ochiai R., Takeda J., Champagnat J., Denavit-Saubié M., Fortin G., Foutz A.S., Thoby-Brisson M. (2004). Mechanism of Propofol-Induced Central Respiratory Depression in Neonatal Rats. Post-Genomic Perspectives in Modeling and Control of Breathing. Advances in Experimental Medicine and Biology.

[25] Jonsson M.M., Lindahl S.G., Eriksson L.I. (2005). Effect of propofol on carotid body chemosensitivity and cholinergic chemotransduction. Anesthesiology.

[26] Eger E.I., Weiskopft R.B., Eisenkraft J.B. (2002). Physical properties. The Pharmacology of Inhaled Anesthetics.

[27] Eger E.I., Weiskopft R.B., Eisenkraft J.B. (2002). MAC. The Pharmacology of Inhaled Anesthetics.

[28] Ang T.N., Udugama I.A., Mansouri S.S., Taylor M., Burrell R., Young B.R., Baroutian S. (2019). A techno-economic-societal assessment of recovery of waste volatile anaesthetics. Sep. Purif. Technol..

[29] Rotaru L.T., Văruț R.M., Truicu F., Gîrniceanu A., Forțofoiu M., Constantin C. (2022). Myoglobin vs hemoglobin blockade model related smoke gas inhalation—A computational analysis. J. Sci. Arts.

[30] Ermondi G., Vallaro M., Goetz G., Shalaeva M., Caron G. (2020). Updating the portfolio of physicochemical descriptors related to permeability in the beyond the rule of 5 chemical space. Eur. J. Pharm. Sci..

[31] Allec S.I., Sun Y., Sun J., Chang C.A., Wong B.M. (2019). Heterogeneous CPU+GPU-Enabled Simulations for DFTB Molecular Dynamics of Large Chemical and Biological Systems. J. Chem. Theory Comput..

[32] Nayim S., Sukanya C., Mohd A., Abdullah A., Dasarath M. (2022). Identification of 4-acrylamido-N-(pyridazin-3-yl)benzamide as anti-COVID-19 compound: A DFTB, molecular docking, and molecular dynamics study. RSC Adv..

[33] Chavez L.O., Leon M., Einav S., Varon J. (2016). Beyond Muscle Destruction: A Systematic Review of Rhabdomyolysisfor clinical practice. Crit. Care.

[34] Walsh D.W., Eckstein M. (2004). Hydrogen cyanide in fire smoke: An underappreciated threat. Emerg. Med. Serv..

[35] Katritzky A.R., Jain R., Petrukhin R., Denisenko S., Schelenz T. (2001). QSAR Correlations of the Algistatic Activity of 5-Amino-1-Aryl-1 H-Tetrazoles. SAR&QSAR Environ. Res..

[36] Cimpoesu D., Corlade-Andrei M., Popa T.O. (2019). Cardiac Arrest in Special Circumstances-Recent Advances in Resuscitation. Am. J. Ther..

[37] Eckstein M., Maniscalco P.M. (2006). Focus on Smoke Inhalation—The Most Common Cause of Acute Cyanide Poisoning. Prehospital Disaster Med..

[38] Goh S.H., Tiah L., Lim H.C., Ng E.K. (2006). Disaster Preparedness: Experience From a Smoke Inhalation Mass Casualty Incident. Eur. J. Emerg. Med..

[39] Woodson L.C. (2009). Diagnosis and Grading of Inhalation Injury. J. Burn. Care Res..

[40] Lawson-Smith P., Jansen E.C., Hilsted L. (2010). Effect of Hyperbaric Oxygen Therapy on whole blood cyanide concentrations in carbon monoxide intoxicated patients from fire accidents. Scand. J. Trauma Resusc. Emerg. Med..

[41] Ronzani M., Woyke S., Mair N., Gatterer H., Oberacher H., Plunser D., Haller T., Ströhle M., Rugg C. (2022). The effect of desflurane, isoflurane and sevoflurane on the hemoglobin oxygen dissociation curve in human blood samples. Sci. Rep..

[42] Edgington T.L., Muco E., Maani C.V. (2023). Sevoflurane.

[43] Khan Z.H., Nabavian O., Mahdi A. (2017). The Effects of Volatile Anesthetic Agents on the Normal Physiological Functions (Indices) of the Cardiovascular System. Arch. Anesth. Crit. Care.

[44] Hao X., Ou M., Li Y., Zhou C. (2021). Volatile anesthetics maintain tidal volume and minute ventilation to a greater degree than propofol under spontaneous respiration. BMC Anesth..

[45] White P.F., Eshima R.W., Maurer A., King T., Lin B.K., Heavner J.E., Bogetz M.S., Kaye A.D. (2003). A comparison of airway responses during desflurane and sevoflurane administration via a laryngeal mask airway for maintenance of anesthesia. Anesth. Analg..

[46] Pappas A.L., Sukhani R., Lurie J., Pawlowski J., Sawicki K., Corsino A. (2001). Severity of airway hyperreactivity associated with laryngeal mask airway removal: Correlation with volatile anesthetic choice and depth of anesthesia. J. Clin. Anesth..

[47] Eger E.I. (2004). Characteristics of anesthetic agents used for induction and maintenance of general anesthesia. Am. J. Health Syst. Pharm..

[48] Guerrero-Orriach J.L., Carmona-Luque M.D., Gonzalez-Alvarez L. (2022). Heart Failure after Cardiac Surgery: The Role of Halogenated Agents, Myocardial Conditioning and Oxidative Stress. Int. J. Mol. Sci..

[49] Yildirim H., Adanir T., Atay A., Katircioğlu K., Savaci S. (2021). The effects of sevoflurane, isoflurane and desflurane on QT interval of the ECG. Eur. J. Anaesthesiol..

[50] Yoon J., Baik J., Cho M.S., Jo J.Y., Nam S., Kim S.H., Ku S., Choi S.S. (2021). Arrhythmia incidence and associated factors during volatile induction of general anesthesia with sevoflurane: A retrospective analysis of 950 adult patients. Anaesth. Crit. Care Pain Med..

[51] Sahu D.K., Kaul V., Parampill R. (2011). Comparison of isoflurane and sevoflurane in anaesthesia for day care surgeries using classical laryngeal mask airway. Indian. J. Anaesth..

[52] De Hert S.G., Cromheecke S., ten Broecke P.W., Mertens E., De Blier I.G., Stockman B.A., Rodrigus I.E., Van der Linden P.J. (2003). Effects of propofol, desflurane, and sevoflurane on recovery of myocardial function after coronary surgery in elderly high-risk patients. Anesthesiology.

[53] Van Allen N.R., Krafft P.R., Leitzke A.S., Applegate R.L., Tang J., Zhang J.H. (2012). The role of Volatile Anesthetics in Cardioprotection: A systematic review. Med. Gas. Res..

[54] Bienengraeber M.W., Weihrauch D., Kersten J.R., Pagel P.S., Warltier D.C. (2005). Cardioprotection by volatile anesthetics. Vasc. Pharmacol..

[55] Zaugg M., Lucchinetti E., Spahn D.R., Pasch T., Schaub M.C. (2022). Volatile anesthetics mimic cardiac preconditioning by priming the activation of mitochondrial K (ATP) channels via multiple signaling pathways. Anesthesiology.

[56] Erturk E. (2014). Ischemia-reperfusion injury and volatile anesthetics. BioMed Res. Int..

[57] Kazanci D., Unver S., Karadeniz U., Iyican D., Koruk S., Yilmaz M.B., Erdemli O. (2009). A comparison of the effects of desflurane, sevoflurane and propofol on QT, QTc, and P dispersion on ECG. Ann. Card. Anaesth..

[58] Chen S., Lotz C., Roewer N., Broscheit J.A. (2018). Comparison of volatile anesthetic-induced preconditioning in cardiac and cerebral system: Molecular mechanisms and clinical aspects. Eur. J. Med. Res..

[59] Zhou Y., Peng D.D., Chong H., Zheng S.Q., Zhu F., Wang G. (2019). Effect of isoflurane on myocardial ischemia-reperfusion injury through the p38 MAPK signaling pathway. Eur. Rev. Med. Pharmacol. Sci..

[60] Bignami E., Guarneri M., Pieri M., De Simone F., Rodriguez A., Cassara L., Zangrillo A. (2017). Volatile anaesthetics added to cardiopulmonary bypass are associated with reduced cardiac troponin. Perfusion.

[61] Piriou V., Chiari P., Lhuillier F., Bastien O., Loufoua J., Raisky O., David J.S., Ovize M., Lehot J.J. (2002). Pharmacological preconditioning: Comparison of desflurane, sevoflurane, isoflurane and halothane in rabbit myocardium. Br. J. Anaesth..

[62] Wu W., Zhou X., Liu P., Fei W., Li L., Yun H. (2014). Isoflurane reduces hypoxia/reoxygenation-induced apoptosis and mitochondrial permeability transition in rat primary cultured cardiocytes. BMC Anesth..

[63] Rivenes S.M., Lewin M.B., Stayer S.A., Bent S.T., Schoenig H.M., McKenzie E.D., Fraser C.D., Andropoulos D.B. (2001). Cardiovascular Effects of Sevoflurane, Isoflurane, Halothane, and Fentanyl–Midazolam in Children with Congenital Heart Disease: An Echocardiographic Study of Myocardial Contractility and Hemodynamics. Anesthesiology.

[64] Cross M., Plunkett E. (2014). The Meyer–Overton Hypothesis. Physics, Pharmacology and Physiology for Anaesthetists: Key Concepts for the FRCA.

[65] Stopfkuchen-Evans M., Bolanos-Diaz L.M., Djalali A.G., Philip B.K., Vacanti C., Segal S., Sikka P., Urman R. (2011). Inhalation anesthetics. Essential Clinical Anesthesia.

[66] Moppett I. (2008). Inhalational anaesthetics. Anesth. Intensive Care Med..

[67] Nielsen J.E. (2007). Analysing the pH-dependent properties of proteins using pK(a) calculations. J. Mol. Graph. Model..

[68] Gunner M.R., Nicholls A., Honig B. (1996). Electrostatic potentials in *Rhodopseudomonas viridis* reaction centers: Implications for the driving force and directionality of electron transfer. J. Phys. Chem..

[69] Talley K., Ng C., Shoppell M., Kundrotas P., Alexov E. (2008). On the electrostatic component of protein to protein binding free energy. PMC Biophys..

[70] Brock K., Talley K., Coley K., Kundrotas P., Alexov E. (2007). Optimization of electrostatic interactions in protein-protein complexes. Biophys. J..

[71] Kundrotas P.J., Alexov E. (2006). Electrostatic properties of protein-protein complexes. Biophys. J..

[72] Mitra R.C., Zhang Z., Alexov E. (2011). In silico modeling of pH-optimum of protein-protein binding. Proteins.

[73] Aguilar B., Anandakrishnan R., Ruscio J.Z., Onufriev A.V. (2010). Statistics and physical origins of pK and ionization state changes upon protein-ligand binding. Biophys. J..

[74] Mitra R., Shyam R., Mitra I., Miteva M.A., Alexov E. (2008). Calculating the protonation states of proteins and small molecules: Implications to ligand-receptor interactions. Curr. Comput.-Aided Drug Design..

[75] Petukh M., Stefl S., Alexov E. (2013). The role of protonation states in ligand-receptor recognition and binding. Curr. Pharm. Des..

[76] Lapostolle F., Fuilla C., Petit M., Lambert Y. (2010). Prise en charge de l’intoxication par les cyanures lors de l’inhalation de fumées d’incendie. Rev. SAMU.

[77] Gilson M.K., Zhou H.X. (2007). Calculation of Protein-Ligand Binding Affinities. Annu. Rev. Biophys. Biomol. Struct..

[78] Martínez-Rosell G., Giorgino T., De Fabritiis G. (2017). PlayMolecule ProteinPrepare: A Web Application for Protein Preparation for Molecular Dynamics Simulations. J. Chem. Inf. Model..

[79] White C.W., Martin J.G. (2010). Chlorine gas inhalation: Human clinical evidence of toxicity and experience in animal models. Proc. Am. Thorac. Soc..

[80] Protein Data Bank Archive (PDB). https://www.rcsb.org/pdb/home/home.do.

[81] ModRefiner Software. https://zhanggroup.org/ModRefiner/.

[82] Morris G.M., Huey R., Lindstrom W., Sanner M.F., Belew R.K., Goodsell D.S., Olson A.J. (2009). AutoDock4 and AutoDockTools4: Automated docking with selective receptor flexibility. J. Comput. Chem..

[83] (2017). The PyMOL Molecular Graphics System.

[84] BIOVIA Dassault Systèmes (2019). BIOVIA Workbook, Release 2017; BIOVIA Pipeline Pilot, Release 2017.

[85] Abraham M.J., Murtola T., Schulz R., Páll S., Smith J.C., Hess B., Lindahl E. (2015). GROMACS: High performance molecular simulations through multi-level parallelism from laptops to supercomputers. SoftwareX.

